# Mitochondrial Dysfunction: A Novel Potential Driver of Epithelial-to-Mesenchymal Transition in Cancer

**DOI:** 10.3389/fonc.2017.00295

**Published:** 2017-12-01

**Authors:** Flora Guerra, Nicoletta Guaragnella, Arnaldo A. Arbini, Cecilia Bucci, Sergio Giannattasio, Loredana Moro

**Affiliations:** ^1^Department of Biological and Environmental Sciences and Technologies (DiSTeBA), Università del Salento, Lecce, Italy; ^2^Institute of Biomembranes, Bioenergetics and Molecular Biotechnologies, National Research Council, Bari, Italy; ^3^Department of Pathology, NYU Langone Medical Center, New York, NY, United States

**Keywords:** epithelial-to-mesenchymal transition, mitochondrial dysfunction, mitochondrial DNA, mitochondrial retrograde signaling, metastasis

## Abstract

Epithelial-to-mesenchymal transition (EMT) allows epithelial cancer cells to assume mesenchymal features, endowing them with enhanced motility and invasiveness, thus enabling cancer dissemination and metastatic spread. The induction of EMT is orchestrated by EMT-inducing transcription factors that switch on the expression of “mesenchymal” genes and switch off the expression of “epithelial” genes. Mitochondrial dysfunction is a hallmark of cancer and has been associated with progression to a metastatic and drug-resistant phenotype. The mechanistic link between metastasis and mitochondrial dysfunction is gradually emerging. The discovery that mitochondrial dysfunction owing to deregulated mitophagy, depletion of the mitochondrial genome (mitochondrial DNA) or mutations in Krebs’ cycle enzymes, such as succinate dehydrogenase, fumarate hydratase, and isocitrate dehydrogenase, activate the EMT gene signature has provided evidence that mitochondrial dysfunction and EMT are interconnected. In this review, we provide an overview of the current knowledge on the role of different types of mitochondrial dysfunction in inducing EMT in cancer cells. We place emphasis on recent advances in the identification of signaling components in the mito-nuclear communication network initiated by dysfunctional mitochondria that promote cellular remodeling and EMT activation in cancer cells.

## Introduction

Mitochondria are the cell powerhouse, on which amino acid, nucleic acid, lipid, and iron–sulfur cluster metabolic pathways converge. During the last decade, mitochondria have been recognized as key players in several aspects of cancer biology, including cancer development, metastasis, and drug resistance (1, 2), due to their central role as receivers, integrators, and transmitters of intracellular signals regulating various processes (3). Mitochondria are highly dynamic organelles whose biogenesis and functions, depending on cellular needs, is under tight nuclear control, through the so-called anterograde regulation, which allows mitochondria adaptation to the ever-changing cellular milieu (4). Only 1% of mitochondrial proteins are encoded by mitochondrial DNA (mtDNA), with all the others encoded by the nuclear genome, including proteins involved in mtDNA replication and transcription, such as mitochondrial single-stranded DNA-binding protein (mtSSB or SSBP1), transcription factor A of mitochondria (TFAM), and mitochondrial DNA polymerase γ (POLG) (5). When cells require enhanced mitochondrial function, anterograde transcriptional regulation of mitochondrial biogenesis is mediated by a set of transcription factors whose activity is regulated by the PPARγ co-activator 1 family members (4).

**Figure 1:**
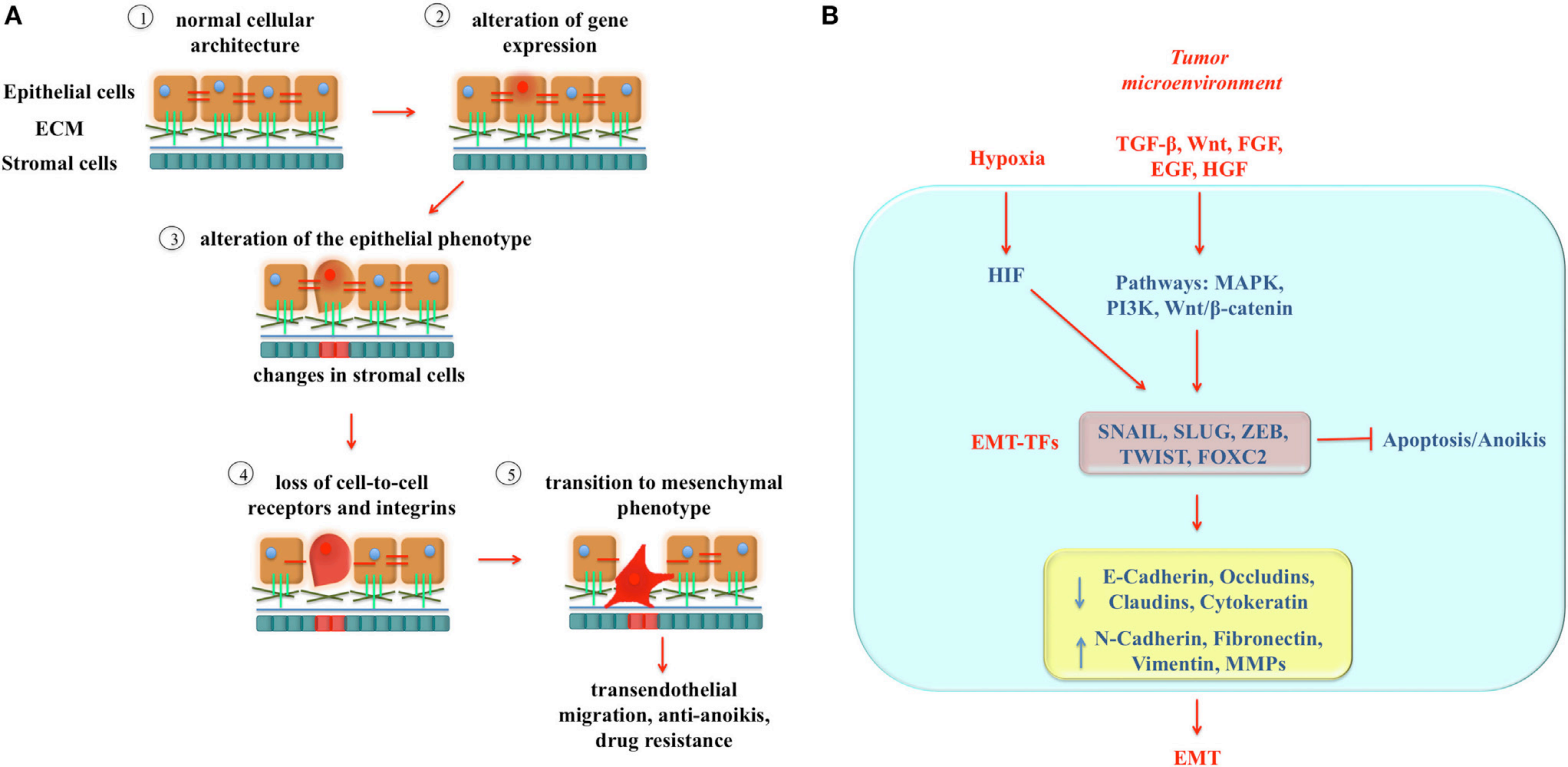
The mechanism of epithelial-to-mesenchymal transition (EMT). **(A)** Cellular changes associated with EMT. Epithelial tumor cells are shown in light brown, and stromal cells are shown in cyan. EMT begins with alterations in gene expression of epithelial cancer cells (step 2) that determine loss of the epithelial phenotype accompanied by alterations in nearby stromal cells (shown as a shift of stromal cell color from blue to red) (step 3). Loss of cell-to-cell attachment receptors and integrins occurs and continues to step 4 and beyond. EMT allows the cells to increase their invasiveness determining degradation of extracellular matrix (ECM) proteins, cytoskeleton reconstruction, extravasation, angiogenesis, as well as anoikis and drug resistance (step 5). **(B)** The regulatory network of EMT. Some important extracellular molecules in the tumor microenvironment, such as TGF-β, HGF, FGF, EGF, and Wnt bind to their respective receptors to induce activation of intracellular pathway, such as MAPK, PI3K, and Wnt/β-catenin. In turn, they regulate induction of EMT-inducing transcription factors (EMT-TFs), including SNAIL, SLUG, ZEB, TWIST, and FOXC2, which are responsible for molecular and physical changes occurring during EMT. Also hypoxia contributes to trigger EMT and participates in the EMT regulatory network through activation of HIFs.

Epithelial-to-mesenchymal transition (EMT) is a complex transdifferentiation process that allows epithelial cancer cells to transiently acquire a predominantly mesenchymal phenotype (6, 7). EMT is characterized by loss of epithelial cell polarity and cell–cell/cell–extracellular matrix contacts, supported by concomitant changes in stromal cells, that enable some tumor cells to migrate out of the primary tumor, cross the basement membrane barriers, and intravasate into the blood stream (8, 9) (Figure 1A). These circulating tumor cells (CTCs) become sources of metastasis at distant sites as the “seeds” in Paget’s “seed and soil” theory (10). EMT requires a complex cellular reprogramming that may render the cells resistant to therapies designed against the primary tumor (11, 12) and has been connected with cancer cell stemness properties (6, 13, 14).

The mutual interplay between EMT and mitochondrial metabolism in cancer has been recently highlighted (15–17). In this relationship, mitochondrial metabolic alterations can drive EMT or, else, EMT activation can fine-tune cancer cell metabolism by affecting the expression of metabolic genes. Mitochondrial dysfunction has been widely implicated in cancer development and progression [for a recent review, see Ref. (2)]. The precise mechanisms underlying mitochondrial dysfunction are multiple and may involve deregulated autophagic processes, unbalance in reactive oxygen species (ROS) homeostasis, mutations in oxidative phosphorylation (OXPHOS) complexes, electron transport chain (ETC), or Krebs’ cycle (TCA) enzymes. Despite the heterogeneity of the mechanisms, EMT induction has been described as one of the endpoint phenotypes in many epithelial tumor cells affected by mitochondrial dysfunction. In this review, we describe how dysregulation of the mitochondrial metabolism and genetics may promote EMT in cancer cells.

## EMT in Cancer

Epithelial-to-mesenchymal transition has been initially described as a physiological process occurring at different stages of the embryonic development (type I EMT) (18). Type II EMT occurs in wound healing and fibrosis (18). Type III EMT is associated with cancer progression (18) and is the focus of this review.

Epithelial-to-mesenchymal transition is a multistep process that involves several molecular changes, including downregulation of the epithelial markers E-cadherin, claudins, desmosomes, and occludins (key components of intercellular junctions) as well as upregulation of the mesenchymal markers N-cadherin, vimentin, and fibronectin, thus fostering motility and invasion (19) (Figure 1B). These changes are orchestrated by transcription factors known as EMT-inducing transcription factors (EMT-TFs), which include TWIST1 and TWIST2, SNAIL 1, SNAIL 2 (SLUG), ZEB1, and ZEB2 as well as non-canonical EMT-TFs such as KLF8, FOXC2, and GSC. EMT-TFs regulate directly or indirectly the expression of adhesive factors and can also induce the expression of matrix metalloproteinases (MMPs), which degrade the basement membrane facilitating invasion and intravasation. Some extracellular factors, such as Wnt, TGF-β, EGF, FGF, and HGF can drive EMT by activating different signaling pathways (MAPK, Wnt/β-catenin, and PI3K) thus promoting the expression of EMT-TFs (20). In addition, tumor hypoxia is considered one of the possible triggers of EMT by inducing hypoxia-inducible transcription factors, e.g., HIF-1α and HIF-2α, which regulate the hypoxic response by modulating the expression of EMT-TFs (21, 22) (Figure 1B).

The pro-metastatic role of EMT-TFs has been extensively demonstrated [for a review, see Ref. (23)]. For example, using genetic mouse models of breast cancer, Tran et al. (24) demonstrated that transient expression of SNAIL 1 in breast tumors was sufficient to increase metastasis. Ectopic expression of TWIST1 in Twist1-negative breast cancer cells also induces EMT and cancer stem cell-like features, including expression of the stem-cell marker CD44 (13, 25–27), suggesting that EMT and acquisition of stemness capacity may be part of the same pathway. Besides promoting migration, invasion and cancer stem-cell properties, EMT would also facilitate survival of CTCs in the peripheral system by inhibiting anoikis as well as apoptosis triggered by chemotherapy or radiotherapy (28, 29). Of note, EMT induction is also regulated by changes in the expression of splicing factors (30): suppression of epithelial-specific splicing proteins (ESPR) is an indicator of the EMT process (31). In addition, identification of epigenetic changes and microRNAs as potent EMT regulators adds further complexity to the regulatory network governing EMT (32, 33).

## Mitochondrial Dysfunction and EMT

Mitochondrial dysfunction has been associated with increased invasiveness, metastatic potential, and drug resistance of cancer cells (2, 34–37). The mechanisms contributing to mitochondrial dysfunction may be multiple and may occur at the level of mtDNA- or nuclear-encoded mitochondrial proteins. In the next paragraphs, we will summarize current knowledge on factors promoting mitochondrial dysfunction that has been implicated in EMT induction in cancer cells.

### Mutations/Changes in Expression of Nuclear-Encoded Mitochondrial Metabolic Enzymes

Mutations in the TCA cycle enzymes fumarate hydratase (FH), isocitrate dehydrogenase (IDH), and succinate dehydrogenase (SDH) have long been recognized as oncogenic but only recently, they have been associated with EMT activation.

Fumarate hydratase mutations suppress conversion of fumarate to malate and cause hereditary leiomyomatosis and highly aggressive renal cell cancer able to metastasize at an early stage even when the primary tumor is still very small (38). Accumulation of fumarate in FH-deficient cells would promote EMT through an epigenetic mechanism: fumarate suppresses the antimetastatic miRNA cluster mir-200ba429 by inhibiting demethylation of a regulatory region, thus resulting in expression of EMT-TFs (39). This novel mechanism provides a rationale to explain the aggressive nature of FH-mutated tumors.

Isocitrate dehydrogenase promotes oxidative decarboxylation of isocitrate to α-ketoglutarate. Mutations in IDH1/2 isoforms are common in oligodendrogliomas and astrocytomas and have been also found in leukemia, melanomas, prostate, colon, and lung cancers (40). Mutant IDHs are neomorphic and catalyze the transformation of α-ketoglutarate to 2-hydroxyglutarate, an oncometabolite that has been shown to induce EMT and to be associated with the presence of distant metastasis in colorectal cancer (41). The oncometabolite 2-hydroxyglutarate, an inhibitor of Jumonji-family histone demethylase, would induce EMT by increasing the trimethylation of H3K4 in the promoter of the *ZEB1* gene, thus increasing the expression of ZEB1, a master regulator of EMT (41).

Succinate dehydrogenase is another TCA cycle enzyme involved in EMT. It catalyzes the conversion of succinate to fumarate and loss-of-function SDH mutations predispose to hereditary pheochromocytoma, paraganglioma, gastrointestinal stromal tumor, and renal cell carcinoma (42). In metastatic pheochromocytomas and paragangliomas, mutations in the SDHB subunit are associated with activation of SNAIL and SLUG as a result of epigenetic remodeling due to hypermethylation of promoter CpG islands (43, 44). Focal deletions of SDHB have been also identified in serous ovarian (45) and colorectal (46) cancer and have been shown to promote EMT through an epigenetic mechanism.

Finally, a combined RNAseq and metabolomics profiling of different solid cancers has shown that downregulation of mitochondrial proteins, particularly those involved in OXPHOS, correlates with poor clinical prognosis across different cancer types and is associated with an EMT gene signature (47). Consistently, loss of OXPHOS genes was observed in metastatic cancer cell lines and in metastatic melanoma and renal cancer specimens. OXPHOS was downregulated in about 60% of low-survival patients, with subunits of Complex I and IV of the ETC being the most affected. In cancers exhibiting OXPHOS downregulation, EMT was the most upregulated cellular program, suggesting a causal role of mitochondrial dysfunction in EMT induction, and, consequently, in cancer aggressiveness and poor outcome.

### mtDNA Modifications

Mutations in mtDNA-encoded proteins also contribute to mitochondrial dysfunction by directly affecting the ETC/OXPHOS system. Until a few years ago, mtDNA was believed to be very susceptible to damage because of absence of DNA repair systems. Nowadays, it is widely accepted that both yeast and mammalian mitochondria are equipped with almost all known nuclear DNA repair pathways, including base excision repair, mismatch repair, single-strand break repair, and possibly non-homologous end joining and homologous recombination [for details, see Ref. (48, 49)]. Despite the presence of DNA repair systems, the mtDNA mutation rate is considerably higher than nuclear DNA, due also to the close proximity of mtDNA to ROS-generating sites. Accumulation of mtDNA mutations has been detected in several cancer types and has been associated with metastatic progression and/or chemoresistance (2, 50–52). In 2008, Ishikawa et al. (53) demonstrated that the mtDNA mutation G13997A in the NADH dehydrogenase (ND) subunit 6 gene promotes metastasis through an ROS-dependent mechanism. Other mtDNA mutations, such as C12084T and A13966G affecting ND4 and ND5, respectively, confer a metastatic phenotype to breast cancer cells but in an ROS-independent manner (54). Another mtDNA mutation affecting ND3 (A10398G) has been detected selectively in bone metastasis of 7/10 prostate cancer patients, suggesting that the A10398G mtDNA mutation may confer a selective advantage to prostate cancer cells to colonize the bone metastatic sites (55). Frequent mtDNA mutations in Complex I genes have been detected in both benign and malignant oncocytic thyroid tumors (56, 57). Intriguingly, oncocytic thyroid carcinomas, also known as Hurthle cell carcinomas, are more aggressive than non-oncocytic thyroid cancers (58, 59), suggesting a potential role of mtDNA mutations in acquisition of the aggressive phenotype. However, despite several evidences showing a link between certain mtDNA point mutations and metastasis, it remains to be investigated whether the mechanism involves EMT activation.

**Figure 2:**
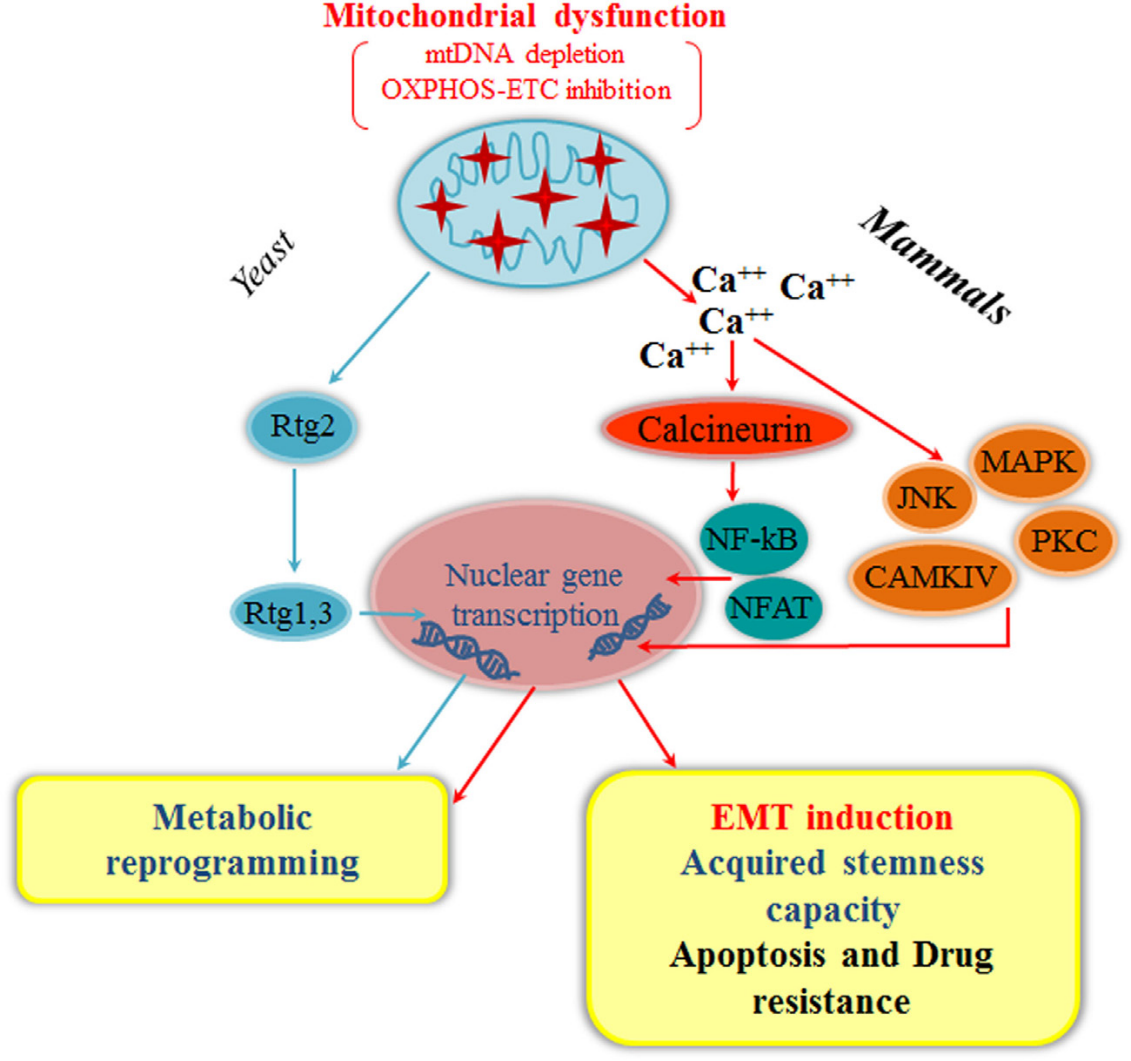
Mitochondrial retrograde signaling and epithelial-to-mesenchymal transition (EMT). Mitochondrial dysfunction, such as mitochondrial DNA (mtDNA) depletion or oxidative phosphorylation (OXPHOS) inhibition, triggers mitochondrial retrograde signaling, which is evolutionary conserved from yeast to mammals. In yeast, Rtg2 regulates the Rtg1,3 translocation into the nucleus eliciting a metabolic reprogramming through the upregulation of specific genes involved in anaplerotic reactions (cyan arrows). In mammals, deregulation in calcium homeostasis due to mitochondrial stress [mtDNA depletion, OXPHOS/electron transport chain (ETC) inhibition] can activate a Ca^++^-dependent retrograde signaling that converges on two possible branches: one mediated by calcineurin for the nuclear translocation of NF-κB or NFAT, and the other directly dependent on activation of Ca^++^-dependent protein kinases, such as PKC, JNK, MAPK, and CAMKIV. These pathways culminate with the activation of different transcription factors that lead to metabolic reprogramming, EMT induction, acquired stemness capacity, apoptosis resistance, and drug resistance (red arrows). Alternative RTG signaling pathways in yeast, *Caenorhabditis elegans*, and mammals are discussed in the text.

Besides single mtDNA mutations, reduction in mtDNA copy number has been reported in several cancer types and has been associated with metabolic reprogramming, increased metastatic potential, chemoresistance, and EMT activation. Different mechanisms have been proposed to explain reduction of mtDNA in cancer cells. Guo et al. (60) reported frequent truncating mutations in the mitochondrial transcription factor TFAM in colorectal cancer cells, which induced mtDNA depletion and apoptosis resistance. A recent study has shown that methylation of the mitochondrial polymerase POLG may also regulate the mtDNA copy number in cancer cells (61). Besides methylation, POLG mutations have been associated with mtDNA depletion in breast cancer tissues (62). Expression changes in other nuclear genes have been reported to affect mtDNA content and induce EMT: for instance, reduced β-catenin levels in basal ErbB2-positive breast cancer cells promote an EMT program through reduction of the mtDNA content, correlated with downregulation of mitochondrial biogenesis transcription factors TFAM and PGC-1α (63). A recent study performed on 207 primary breast tumor specimens shows a direct correlation between low mtDNA content and presence of distant metastasis: patients with ≤350 mtDNA molecules per cell showed a poorer 10-year distant metastasis-free survival compared with patients with> 350 mtDNA molecules per cell (64), suggesting that low mtDNA content might be a prognostic marker for distant metastasis in breast cancer. Reduced mtDNA content has been associated with aggressive features also in other cancer types, including prostate (35, 65, 66) and colorectal (60) cancers, and it has been directly correlated with induction of EMT through activation of mitochondria-to-nucleus signaling (retrograde signaling; Figure 2).

### Mitophagy

Autophagy is the master mechanism of cell homeostasis through which destruction of unnecessary or dysfunctional molecules and organelles occur (67, 68). Withdrawal of nutrients and various stress conditions, such as alterations in glucose metabolism (69, 70), mitochondrial dysfunction, and oxidative stress (71, 72), induce autophagy with the aim of removing damaged macromolecules and organelles and/or to digest cell components to help the cell’s own maintenance (73–76). Being a homeostatic process, autophagy may have a double and opposite role in cancer, behaving as both tumor-promoter and tumor-suppressor depending on cancer cell type and tumorigenic context (77, 78). Cancer cells may indeed activate autophagy to overcome microenvironmental (nutrient deprivation, cell detachment, and hypoxia) or therapeutic (radiotherapy and chemotherapy) stress, thus promoting cancer progression (79, 80).

Mitophagy is a selective form of autophagy that specifically removes dysfunctional mitochondria from the cells. Besides traditional autophagy-related (ATG) proteins, such as LC3 (ATG8) and Beclin1 (ATG6), mitophagy relies upon specific proteins, including the E3 ubiquitin ligase Parkin (PARK2) and mitochondrially targeted PTEN-induced kinase-1 (81, 82). In yeast cells, Atg32, an outer mitochondrial membrane protein, is essential for mitophagy (83–86). Recently, Bcl2-L-13 has been identified as the mammalian homolog of Atg32: it induces mitophagy in Parkin-deficient cells (87), but its role in cancer remains to be investigated. Impaired Parkin activity in mammals has been correlated with cancer progression, suggesting that mitophagy may represent a tumor suppression mechanism (82). On the other hand, Whelan et al. (88) have recently reported that mitophagy supports EMT-mediated conversion of low CD44- to high CD44-expressing keratinocytes through modulation of oxidative stress and Parkin-dependent mitochondrial clearance. In this model, mitophagy was associated with mtDNA depletion, an event known to induce EMT and high-CD44 cell generation in mammary epithelial cells (89). It remains to be established if mitophagy drives EMT-mediated high-CD44 cell generation or is a permissive factor during this process. An independent recent study confirmed a positive role of mitophagy during EMT: Marín-Hernández et al. (90) reported that simultaneous exposure of cancer cells to hypoxia and hypoglycemia results in EMT activation and increased invasiveness, accompanied by activation of mitophagy and impaired mitochondrial functionality.

Taken together, these studies indicate a possible dichotomous nature of the relationship between EMT and mitophagy, which may be ascribed to cell type- and context-dependent factors, but much remains to be investigated.

## Mitochondrial Retrograde Signaling and EMT

Dysfunctional mitochondria can generate a wide range of retrograde responses, i.e., intracellular signals relayed from mitochondria to the nucleus, leading to changes in the expression of nuclear genes for metabolic adjustments and cytoprotection (91–93). The first mitochondrial retrograde signaling was discovered by Butow (94) in yeast *Saccharomyces cerevisiae*. The main positive regulators of mitochondria-to-nucleus in yeast are three retrograde response (RTG) genes: *RTG1* and *RTG3*, encoding for a heterodimeric transcription factor activating RTG target gene expression (95). *RTG2*, coding for a cytoplasmic protein with an N-terminal ATP-binding domain, acts as a sensor of the mitochondrial dysfunction and regulates Rtg1/3p localization (96). RTG genes dynamically interact with other regulators and signaling pathways to elicit a metabolic reprogramming through activation of anaplerotic reactions, supplying intermediates in response to respiratory defects initiated by mtDNA depletion/mutations or disruption of ETC/OXPHOS (97) (Figure 2). Interestingly, *AUP1* encoding for a conserved mitochondrial protein phosphatase required for mitophagy in yeast has been shown to induce the *RTG3*-dependent retrograde signaling pathway (98), suggesting a possible interplay between mitophagy and mitochondrial retrograde signaling.

Another mitochondrial retrograde pathway, induced by mitochondrial proteotoxic stress, was discovered in mammalian cells by the pioneering work of Hoogenraad (99), but its detailed regulation has recently been elucidated in *Caenorhabditis elegans* (100). Disturbance of mitochondrial protein homeostasis and/or an increase in unassembled components initiates an retrograde response named mitochondrial unfolded-protein response (UPR^mt^). The current paradigm suggests that peptides resulting from proteolytic degradation of improperly folded mitochondrial proteins are released from mitochondria. However, mitochondrial import efficiency is reduced during mitochondrial dysfunction, causing ATFS-1, a pivotal transcription factor of the UPR^mt^, to accumulate in the cytosol and subsequently be imported into the nucleus. ATFS-1 in the nucleus regulates a transcriptional response to recover mitochondrial function including induction of mitochondrial proteases and chaperones, ROS detoxifying genes, and metabolic regulators leading to metabolic reprogramming (93, 100). The transcription factor ATF5 was recently identified as the mammalian ortholog of ATFS-1 (101). While a body of literature is already present on the function of ATF5 in cancer biology, notably in the regulation of survival and apoptosis (102, 103), it will be interesting to explore the role of ATF5 in the context of UPR^mt^ and cancer, particularly in EMT regulation and metastasis.

The mitochondrial retrograde signaling is conserved in mammals both in response to energy metabolism impairment and to proteotoxic stress (93, 104). Of the multiple retrograde signaling pathways activated in mammals by mitochondrial dysfunction (91, 105), Ca^++^/calcineurin-mediated retrograde signaling has been involved in EMT activation (105) (Figure 2). Ca^++^ homeostasis strictly depends on mitochondria and its deregulation due to different mitochondrial stresses, such as mtDNA depletion or ETC/OXPHOS inhibition, can elicit an increase in cytosolic Ca^++^ that activates a Ca^++^-dependent retrograde signaling. Depending on cell type and conditions, there are essentially two branches in this pathway: (i) a Ca^++^-calcineurin-mediated retrograde signaling, through the nuclear translocations of transcription factors, NF-κB, NFAT, CREB, and HnRNPA2; (ii) a direct activation of Ca^++^-dependent protein kinases, such as PKC, JNK, MAPK, and CAMKIV (94, 104). Activation of these signaling pathways in epithelial cells converge on the upregulation of genes affecting several cellular functions, including apoptosis resistance, multidrug resistance, invasion, and EMT (66, 89, 106). Mitochondrial dysfunction induced by mtDNA depletion promotes EMT in breast epithelial cells through a calcineurin A-mediated mitochondrial retrograde signaling that triggers transcriptional activation of SLUG, SNAIL, and TWIST, the MMP-9 metalloproteinase, and the mesenchymal markers fibronectin, vimentin, and N-cadherin, with a corresponding decrease in the epithelial marker E-cadherin. In addition, mtDNA-depleted breast cells exhibited loss of the ESPR such as ESPR1, indicative of their mesenchymal phenotype, and expressed stem-cell markers, suggesting generation of cancer stem cells (13) (Figure 2). Of note, mtDNA-depleted cells exhibit also unorganized trajectory and higher mitochondrial fission, characteristic of cells with high metastatic ability (105). The potential link between mitochondrial dysfunction and EMT was also reported in prostate and breast adenocarcinoma cell lines depleted of mtDNA, which acquired a mesenchymal phenotype and showed TGF-β overexpression (107). More recently, mtDNA depletion was shown to induce EMT in hepatocellular carcinoma cells through TGF-β/SMAD/SNAIL signaling (108). In addition, suppression of SSBP1 promoted triple-negative breast cancer cell metastasis through mtDNA depletion, which triggered calcineurin A-mediated mitochondrial retrograde signaling resulting in c-Rel/p50 translocation to the nucleus, increased levels of TGF-β and TGF-β-driven EMT (109).

## Concluding Remarks

Epithelial-to-mesenchymal transition endows cancer cells with the ability to detach from the primary tumor bulk and survive during invasion, dissemination, and metastasis. The observation that mitochondrial dysfunction can drive EMT is important as it unfolds novel therapeutic scenarios: EMT could be potentially blocked by targeting mitochondrial stress-specific EMT marker genes, effectors of the mitochondrial retrograde signaling, specific metabolic enzymes, or metabolism-dependent epigenetic reprogramming, with the aim to limit or prevent cancer metastasis. Several questions, however, remain to be answered. For instance, how and why different types of mitochondrial dysfunction converge on EMT remains a puzzle. It is possible that transient transition to a mesenchymal phenotype may confer a survival advantage to epithelial cancer cells under nutrient or oxygen stress, or in the presence of genetic defects in metabolic enzymes. In this context, EMT would represent a strategy to equip cancer cells with the necessary “armor” (increased survival) and “skills” (increased motility, invasion) to strive while exploring more advantageous metabolic microenvironments. Further studies aimed at understanding the interplay between mitochondrial retrograde signaling pathways and changing microenvironments as well as identifying the molecular determinants of the mito-nuclear network linking mitochondrial dysfunction with EMT activation may provide useful therapeutic targets for treatment and prevention of metastatic cancer.

## Author Contributions

LM and SG designed and outlined structure and contents of the review. FG, NG, AA, CB, SG, and LM contributed to the literature analysis, interpretation, and writing of the review.

## Conflict of Interest Statement

The authors declare that the research was conducted in the absence of any commercial or financial relationships that could be construed as a potential conflict of interest.
